# Mental Health Status of University Healthcare Workers during the COVID-19 Pandemic: A Post–Movement Lockdown Assessment

**DOI:** 10.3390/ijerph17249155

**Published:** 2020-12-08

**Authors:** Luke Sy-Cherng Woon, Hatta Sidi, Nik Ruzyanei Nik Jaafar, Mohammad Farris Iman Leong Bin Abdullah

**Affiliations:** 1 Department of Psychiatry, Universiti Kebangsaan Malaysia Medical Centre, Jalan Yaacob Latif, Bandar Tun Razak, Cheras, Kuala Lumpur 56000, Malaysia; lukewoon@gmail.com (L.S.-C.W.); hattasidi@hotmail.com (H.S.); njruzyanei@gmail.com (N.R.N.J.); 2 Lifestyle Science Cluster, Advanced Medical and Dental Institute, Universiti Sains Malaysia, Kepala Batas 13200, Pulau Pinang, Malaysia

**Keywords:** depression, anxiety, stress, university healthcare workers, COVID-19, post–movement lockdown

## Abstract

This study investigated the prevalence and severity of depression, anxiety, and stress and determined the association between various factors, social support, and depression, anxiety, and stress among university healthcare workers in Malaysia after the government lifted the movement control order (MCO) put in place to curb the coronavirus disease 2019 (COVID-19) pandemic. This online, cross-sectional survey recruited 399 participants from two university hospitals, and they were administered a self-reported questionnaire on demographic, personal, and clinical characteristics, as well as COVID-19-related stressors and coping. In addition, they completed the Multidimensional Scale of Perceived Social Support (MSPSS) to measure perceived social support, as well as the 21-item Depression, Anxiety, and Stress Scale (DASS-21) to assess depression, anxiety, and stress. We found that the prevalence rates of depression, anxiety, and stress were 21.8%, 31.6%, and 29.1%, respectively. Participants with moderate to extremely severe depression, anxiety, and stress made up 13.3%, 25.8%, and 8.1% of the sample, respectively. Being single or divorced, fear of frequent exposure to COVID-19 patients, agreeing that the area of living had a high prevalence of COVID-19 cases, uncertainty regarding the prevalence of COVID-19 cases in the area of living, and a history of pre-existing psychiatric illnesses were associated with higher odds of depression, anxiety, and stress. Conversely, having more than three children and greater perceived friend support were associated with lower odds of depression, anxiety, and stress. The prevalence of depression, anxiety, and stress remained elevated even after the MCO was lifted.

## 1. Introduction

Coronavirus disease 2019 (COVID-19), caused by the previously unknown severe acute respiratory syndrome coronavirus 2 (SARS-CoV-2), began in the city of Wuhan, China, in December 2020 as clusters of mysterious respiratory tract infections [1]. Since then, the infection has rapidly spread throughout China and to other parts of the world. Consequently, the World Health Organization declared COVID-19 a global pandemic on 11 March 2020 [2]. In the Malaysian context, COVID-19 was initially transmitted from travelers to the local population between January and February 2020; this resulted in a massive spread of infection in early March, when a large cluster of infections emerged called the Tablighi Jamaat cluster [3]. A movement control order (MCO) was imposed by the government from 18 March 2020, to 9 June 2020; this prohibited the population from organizing any social activities and gatherings, including cultural, religious, sporting, work-related, and educational activities [4].

Because of the prolonged confinement during movement lockdowns, psychological distress in the population has been reported during infection epidemics; this presents in the form of various symptoms, including low mood, insomnia, stress, anxiety, anger, irritability, and emotional exhaustion [5]. Healthcare workers who provide clinical services are required to work in a high-risk environment. Higher prevalence rates of depression, anxiety, insomnia, obsessive-compulsive and somatization symptoms, and posttraumatic stress symptoms have been reported in medical healthcare workers compared with non-medical personnel. In fact, higher anxiety has been reported to correlate positively with stress among healthcare workers during the COVID-19 pandemic [6,7,8]. Female gender, living in rural areas, being the only child in the family, lower availability of social support, having organic diseases, risk of exposure to COVID-19-positive patients, risk of contracting the disease, worry about a lack of medical supplies, and long working hours are some of the main stressors healthcare workers have experienced during the challenging time of the COVID-19 pandemic. This has placed them at high risk of developing depression, anxiety, and insomnia [6,7,9,10,11]. In addition, a history of having similar physical symptoms to those of COVID-19 infection (e.g., cough, sore throat, lethargy, breathlessness, poor appetite, and myalgia) also predisposes healthcare workers to depression, anxiety, stress, and posttraumatic stress symptoms [12]. In contrast, the availability of protective equipment and strict standard operating procedures to curb the spread of infection in the hospital, recognition of the effort of healthcare workers providing sufficient care for COVID-19 patients, and reduction in reported cases of COVID-19 reduces the risk of psychological impact of the COVID-19 pandemic [9].

Despite the substantial effect of the COVID-19 pandemic on healthcare workers, data regarding COVID-19′s psychological impact on these workers in Malaysia is scarce. Moreover, to the best of our knowledge, data on the mental health status of healthcare workers after the movement lockdown was lifted are also lacking, although the literature has reported that psychological sequelae may persist beyond the movement lockdown [5]. This study was designed to accomplish the following: (1) investigate the prevalence and severity of depression, anxiety, and stress in university healthcare workers and (2) determine the association between various factors (demographic, personal, and clinical characteristics; COVID-19-related stressors; and coping), perceived social support, and depression, anxiety, and stress among university healthcare workers in Malaysia after the movement lockdown was lifted.

## 2. Materials and Methods

### 2.1. Study Design and Participants

This was an online, cross-sectional study conducted using the Google Forms platform. Google Forms is an online survey administration software that allows questionnaires to be posted and completed by targeted subjects online. The collected data can then be automatically entered into a spreadsheet. This online survey was administered from 1 July 2020, to 21 July 2020, starting about three weeks after the MCO ended in Malaysia. The source population was active full-time staff members at the university hospitals of two public universities in Malaysia, namely, Hospital Universiti Sains Malaysia (HUSM) and Universiti Kebangsaan Malaysia Medical Centre (UKMMC). We selected university healthcare workers for this study because data on the psychological impact of the COVID-19 pandemic on this group of healthcare workers are scarce both in Malaysia and globally. Moreover, because of the multitasking nature of the work scope of university medical academic staff, who are required to provide healthcare services and carry out academic duties, it is pivotal to investigate how the COVID-19 pandemic affects their mental health status. The source population included healthcare professionals, academic staff, and allied healthcare staff involved in clinical care in the two university hospitals. HUSM was selected because it is the largest university hospital in the northern region of Peninsular Malaysia, whereas UKMMC was selected because it is a large university hospital in the Klang Valley of Peninsular Malaysia, an urban conglomeration with a population of 8 million people. HUSM has 3000 staff, and UKMMC has 3873 staff. These numbers include healthcare staff involved in clinical care and staff involved in non-clinical duties, such as those who perform administrative work. The proportion of staff who participated in this study was 5.8% of the total number of staff members in both hospitals. The study sample was recruited via snowball sampling because it was conducted online to abide by the new norm of social distancing during the COVID-19 pandemic. With permission obtained from the hospital management authorities, an invitation email to participate in the study was circulated to the academic staff of HUSM and UKMMC. Then, the participants were asked to share the email invitation with other healthcare professionals, academic staff, and allied healthcare staff involved in clinical care in the two university hospitals. The setting of the online survey form was adjusted to prevent repeated submissions from the same participant by activating the “limiting responses to once per person” function in Google Forms. Healthcare workers were eligible to participate in the study if they met the following criteria: (1) age of 18 years and above, (2) active full-time staff members of HUSM and UKMMC involved in clinical care of patients (healthcare professionals, academic staff, and allied healthcare staff), and (3) no history of pre-existing psychotic disorders, bipolar mood disorder, or illicit drug use or alcohol dependence. Details of the study procedures were provided to the participants in the invitation email. Participants were considered to have provided informed consent to participate in the study when they completed and submitted their responses to the online survey. This study was approved by the Medical Research Committee of the Faculty of Medicine, Universiti Kebangsaan Malaysia (UKMPPI/111/8/JEP-2020-370) and the Human Research Ethics Committee of Universiti Sains Malaysia (USM/JEPeM/COVID19-21).

### 2.2. Measures

Data on demographic and personal characteristics, clinical factors, COVID-19-related stressors, and religious coping of the participants were collected by administering a self-reported questionnaire. The questionnaire was constructed based on the information from previous studies on the psychological impact of infection epidemics among the general population, such as SARS and Middle East respiratory syndrome (MERS) [13,14,15,16,17,18,19]. The demographic factors and personal characteristics (e.g., age, gender, marital status, education status, and living arrangements during the pandemic), clinical factors (e.g., pre-existing history of medical illness and psychiatric illness), and epidemic-related stressors (e.g., worrying about family during the pandemic, frustration because of loss of daily routine during the pandemic, feeling stress because annual leave was frozen during the pandemic, average working hours per week during the pandemic, fear of frequent exposure to COVID-19 patients, and having a history of quarantine after coming into close contact with infected patients) found to be associated with psychological complications during the SARS and MERS epidemics in previous studies were included in the self-reported questionnaire. The questionnaire was not fully validated, but it was constructed by a group of experts comprising four psychiatrists, one psychologist, and one public health specialist; this was done based on data from previous studies of the psychological complications during the SARS and MERS epidemics to ensure content validity. In addition, the questionnaire was tested for 20 university healthcare workers online and then assessed for the comprehensiveness of the sentence structures, words, and instructions, as well as its semantic quality and the appropriateness of the duration of administration; this was done to ensure face validity before it was used in this study. The participants were also administered the Malay version of the Multidimensional Scale of Perceived Social Support (MSPSS) to assess the degree of perceived social support, as well as the Malay version of the 21-item Depression, Anxiety, and Stress Scale (DASS-21) to evaluate depression, anxiety, and stress.

#### 2.2.1. Demographic and Personal Characteristics

Data on demographic characteristics, such as age, gender, marital status, education status, and religion, were collected. Personal characteristics, such as the number of children the participant had and living arrangements during the COVID-19 pandemic, were also recorded. The description of all the variables and categorization of the groupings are illustrated in Table 1.

#### 2.2.2. Clinical Factors

We collected data on two clinical factors, which were a pre-existing history of medical illness and a pre-existing history of psychiatric illness. Pre-existing medical illness was documented as a self-reported physician diagnosis of hypertension, diabetes, chronic lung disease, heart disease, endocrine diseases, central nervous system diseases, renal diseases, gynecological and genitourinary tract diseases, or cancer. Pre-existing psychiatric illness involved a self-reported psychiatrist diagnosis of depression and anxiety disorders. The description of all the variables and categorization of the groupings are presented in Table 1.

#### 2.2.3. COVID-19-Related Stressors and Religious Coping

Several stressors related to the COVID-19 pandemic were documented, such as worrying about family during the pandemic, frustration because of loss of daily routine during the pandemic, feeling stress because annual leave was frozen during the pandemic, average working hours per week during the pandemic, fear of frequent exposure to COVID-19 patients, experiencing fear when similar physical symptoms to COVID-19 symptoms developed, living in an area highly prevalent for COVID-19-positive cases, and having a history of quarantine after coming into close contact with COVID-19-positive cases. Data on the perception that religious coping helped in managing stress during the COVID-19 pandemic were also recorded. The description of all the variables and categorization of the groupings are summarized in Table 1.

#### 2.2.4. Perceived Social Support

The perceived social support of the participants was measured using the MSPSS. This self-reported tool evaluates the social support received by an individual in three domains (family, friends, and significant others social support). Each domain consists of four items; hence, the instrument comprises a total of 12 items. Each item is scored on a 7-point Likert scale with a range of scores from 1 to 7. Therefore, each domain registers a cumulative score of 4 to 28. A higher score signifies a greater degree of social support. The MSPSS has demonstrated good internal consistency (Cronbach’s α = 0.88) and test-retest reliability (Cronbach’s α = 0.85) [20]. The validated Malay version of the MSPSS also showed satisfactory internal consistency (Cronbach’s α = 0.89) and test-retest reliability (Cronbach’s *α* = 0.77) [21]. The Malay version of the MSPSS showed excellent internal consistency in this study, with a Cronbach’s α of 0.96. The internal consistency of the three domains of the Malay version of the MSPSS were also excellent, with Cronbach’s α results for family support, friends support, and significant others support domains of 0.92, 0.95, and 0.94, respectively.

#### 2.2.5. Depression, Anxiety, and Stress

The participants’ depression, anxiety, and stress symptoms were assessed using the DASS-21. This self-reported instrument comprises 21 items designated to three subscales, which are the depression, anxiety, and stress subscales. Each subscale consists of seven items, and each item is scored on a Likert scale of 0 to 3. The cumulative score for each subscale is computed by summing the scores for the items and multiplying by two. A higher score indicates more severe symptoms. The cut-off points for a case finding are 9 for depression, 7 for anxiety, and 14 for stress. In addition, the ranges of scores for severity of depression are as follows: (1) mild depression = 10–13, (2) moderate depression = 14–20, (3) severe depression = 21–27, and (4) extremely severe depression = 28–42. The ranges of scores for severity of anxiety are as follows: (1) mild anxiety = 8–9, (2) moderate anxiety = 10–14, (3) severe anxiety = 15–19, and (4) extremely severe anxiety = 20–42. The ranges of scores for severity of stress are as follows: (1) mild stress = 15–18, (2) moderate stress = 19–25, (3) severe stress = 26–33, and (4) extremely severe stress = 34–42 [22]. The Malay version of the DASS-21 has been demonstrated to have acceptable internal consistency, with Cronbach’s α values of 0.75, 0.74, and 0.79 for the depression, anxiety, and stress subscales, respectively. It also exhibits good construct validity [23]. The internal consistency of the Malay version of the DASS-21 subscales in this study was good, where the Cronbach’s α values of the depression, anxiety, and stress subscales were 0.89, 0.85, and 0.92, respectively.

### 2.3. Statistical Analyses

Data analyses were conducted using IBM SPSS Statistics version 26 (SPSS Inc., Chicago, IL, USA). Descriptive statistics were computed for demographic and personal characteristics; clinical factors; COVID-19-related stressors; religious coping; the social support domain scores; and the prevalence and severity of depression, anxiety, and stress. There were no missing values. Categorical variables were presented as frequencies and percentages, while continuous variables were described as the mean and standard deviation (SD). Then, simple logistic regression analyses were applied to determine the individual association between various demographic and personal characteristics, clinical factors, COVID-19-related stressors, religious coping, the social support domain scores (independent variables) and depression, anxiety, and stress (dependent variables) by computing the crude odds ratios (ORs), where absence of depression, anxiety, or stress was coded as 0 (reference) and presence of depression, anxiety, or stress was coded as 1. Then, variables with *p* < 0.1 were entered into multiple logistic regression models to determine their adjusted ORs in terms of predicting depression, anxiety, and stress (dependent variables). Similarly, the absence of depression or anxiety was coded as 0 (reference), whereas the presence of depression or anxiety was coded as 1. The multiple logistic regression model fit was assessed by referring to the Hosmer–Lemeshow test, where *p* > 0.05 indicated model fit. Forward and backward stepwise logistic regression analyses were used to confirm the significant predictors of depression, anxiety, and stress. Statistical significance was set at *p* < 0.05, and all *p*-values were two-sided.

## 3. Results

### 3.1. Participant Characteristics

In total, 399 participants completed the online survey. The demographic and personal characteristics, clinical factors, COVID-19-related stressors, and coping of the participants are summarized in Table 1. More than half the participants were 30–45 years old (*n* = 266, 66.7%), and nearly three-quarters were females (*n* = 292, 73.2%). About three-quarters of the participants were married (*n* = 308, 77.2%), and most had up to tertiary education (*n* = 354, 88.7%). Most lived with family during the COVID-19 pandemic (*n* = 350, 87.7%). About half the participants had one to three children (*n* = 201, 50.4%), while almost one-quarter had more than three children (*n* = 88, 22.0%).

Assessment of the COVID-19-related stressors revealed that only a minority of the participants worried about their family (*n* = 45, 11.3%), but almost one-third felt frustrated because of loss of daily routine as a consequence of social distancing (*n* = 125, 31.3%). More than half the participants were bothered by fear when they developed physical symptoms that were similar to COVID-19 symptoms, such as cough, fever, and flu (*n* = 239, 59.9%), while only one-fifth of them agreed that their area of living was highly prevalent for COVID-19-positive cases (*n* = 80, 20.1%). Regarding work-related stressors during the COVID-19 pandemic, only a minority of participants experienced stress because of their annual leave being frozen as part of the hospital management’s strategy to strengthen the clinical service during the COVID-19 pandemic (*n* = 67, 16.8%), and only a small proportion were quarantined for 14 days because of being in close contact with COVID-19-positive cases (*n* = 31, 7.8%). However, almost half experienced fear resulting from frequent exposure to COVID-19 patients as part of their work (*n* = 192, 48.1%). Most participants agreed that religion helped them cope with stress during the COVID-19 pandemic (*n* = 344, 86.2%). Assessment of the clinical factors showed that more than one-fifth of the participants had pre-existing medical illness (*n* = 107, 26.8%), but only a minority had pre-existing psychiatric illness (*n* = 11, 2.8%).

The perceived social support and psychological characteristics of the participants are illustrated in Table 2. Based on the DASS-21 scores, 21.8% of participants had depression, with 8.5% exhibiting mild depression, 9.8% moderate depression, and 3.8% severe to extremely severe depression. In addition, 31.6% of the participants had anxiety, where 5.8% exhibited mild anxiety, 18.0% moderate anxiety, and 7.8% severe to extremely severe anxiety. The proportion of participants with stress was 29.1%, where 21.1% exhibited mild stress, 4.8% moderate stress, and 3.3% severe to extremely severe stress. The mean scores for the three domains of the MSPSS were 22.51 (SD = 4.77) for the family support domain, 20.96 (SD = 4.56) for the friends support domain, and 22.05 (SD = 5.39) for the significant others support domain.

### 3.2. The Associations Among Clinical Factors, COVID-19-Related Stressors, and Coping, Social Support, and Depression

Table 3 shows the association between various demographic, personal, and clinical factors; COVID-19-related stressors; and coping, social support, and depression among the participants. Simple logistic regression analyses indicated that several factors were significantly associated with depression (*p* < 0.1), such as marital status, number of children, living arrangement during COVID-19, history of pre-existing psychiatric illnesses, worrying about family during the COVID-19 pandemic, frustration because of loss of daily routine during the MCO, fear from frequent exposure to COVID-19 patients, agreement that the area of living had a high prevalence of COVID-19 cases, and levels of family, friends, and significant others social support. These variables were included in the multiple logistic regression model. However, the model revealed that there were only two COVID-19-related stressors, fear from frequent exposure to COVID-19 patients (adjusted OR = 3.735, 95% confidence interval [CI] = 1.208–11.551, *p* = 0.022) and agreement that the area of living had a high prevalence of COVID-19 cases (adjusted OR = 2.782, 95% CI = 1.275–6.068, *p* = 0.010), which predicted a significantly higher likelihood of depression. Having more than three children was protective against depression (adjusted OR = 0.237, 95% CI = 0.061–0.921, *p* = 0.038). Moreover, none of the clinical factors precipitated depression, and none of the social support modalities were protective against depression. 

The multiple logistic regression model reported a Nagelkerke *R*^2^ of 0.315 (*p* < 0.001), and the Hosmer–Lemeshow goodness-of-fit test (χ^2^ = 6.585, *df* = 8, *p* = 0.582) indicated good model fit. The forward and backward stepwise logistic regression analyses confirmed that experiencing fear from frequent exposure to COVID-19 patients and agreement that the area of living had a high prevalence of COVID-19 cases significantly predicted a higher likelihood of depression. While having more than three children was protective against depression. Other independent variables were not significantly associated with depression.

### 3.3. Associations among Clinical Factors, COVID-19-Related Stressors, and Coping, Social Support, and Anxiety

Table 4 summarizes the association among various demographic, personal, and clinical factors; COVID-19-related stressors; and coping, social support, and anxiety for the participants. Simple logistic regression analyses indicated that several factors were significantly associated with anxiety (*p* < 0.1), including age, marital status, number of children, history of pre-existing psychiatric illnesses, frustration because of loss of daily routine during the MCO, feeling stress because of annual leave being frozen during the COVID-19 pandemic, fear of frequent exposure to COVID-19 patients, having fear when developing similar physical symptoms to those that arise in COVID-19 infection, agreement that the area of living had a high prevalence of COVID-19 cases, and levels of family, friends, and significant others social support. These variables were included in the multiple logistic regression model. The model revealed that one demographic factor (being single/divorced; adjusted OR = 3.964, 95% CI = 1.327–11.838, *p* = 0.014) and two COVID-19-related stressors (experiencing fear because of frequent exposure to COVID-19 patients; adjusted OR = 4.328, 95% CI = 1.515–12.361, *p* = 0.006; and not knowing whether the area of living was highly prevalent for COVID-19-positive cases; adjusted OR = 2.514, 95% CI = 1.085–5.827, *p* = 0.032) significantly predicted a higher likelihood of anxiety. In contrast, higher perceived social support from friends was the only protective factor against anxiety (adjusted OR = 0.893, 95% CI = 0.819–0.973, *p* = 0.009). The multiple logistic regression model reported a Nagelkerke *R*^2^ of 0.289 (*p* < 0.001), and the Hosmer–Lemeshow goodness-of-fit test (χ^2^ = 11.470, *df* = 8, *p* = 0.176) indicated good model fit. The forward and backward stepwise logistic regression analyses confirmed that being single/divorced, experiencing fear from frequent exposure to COVID-19 patients, and not knowing whether the area of living was highly prevalent for COVID-19-positive cases significantly predicted a higher likelihood of anxiety. Higher perceived social support from friends was protective against anxiety. None of the other variables were significantly associated with anxiety.

### 3.4. Associations among Clinical Factors, COVID-19-Related Stressors, and Coping, Social Support, and Stress

Table 5 illustrates the association among various demographic, personal, and clinical factors; COVID-19-related stressors; and coping, social support, and stress for the participants. Simple logistic regression analyses showed that several factors were significantly associated with stress (*p* < 0.1), which included marital status, number of children, history of pre-existing medical and psychiatric illnesses, worrying about family during the COVID-19 pandemic, frustration because of loss of daily routine during the MCO, fear of frequent exposure to COVID-19 patients, fear when developing similar physical symptoms to those of COVID-19 infection, agreement that the area of living had a high prevalence of COVID-19 cases, quarantine for 14 days because of being a close contact of a COVID-19-positive patient, and the levels of family, friends, and significant others social support. Nevertheless, the multiple logistic regression model indicated that only one COVID-19-related stressor (experiencing fear because of frequent exposure to COVID-19 patients; adjusted OR = 2.540, 95% CI = 1.014–6.364, *p* = 0.047) and one clinical factor (a history of pre-existing psychiatric illnesses; adjusted OR = 13.382, 95% CI = 1.248–143.483, *p* = 0.032) significantly predicted a higher likelihood of stress. A higher level of social support from friends was the only factor that was protective against stress (adjusted OR = 0.909, 95% CI = 0.834–0.990, *p* = 0.029). The multiple logistic regression model reported a Nagelkerke *R*^2^ of 0.249 (*p* < 0.001), and the Hosmer–Lemeshow goodness-of-fit test (χ^2^ = 4.781, *df* = 8, *p* = 0.781) indicated good model fit. The forward and backward stepwise logistic regression analyses confirmed that experiencing fear because of frequent exposure to COVID-19 patients and a history of pre-existing psychiatric illnesses significantly predicted a higher likelihood of stress. Higher perceived social support from friends was protective against stress; none of the other variables were significantly associated with stress.

## 4. Discussion

This study investigated the prevalence and severity of depression, anxiety, and stress among university healthcare and allied healthcare workers after the MCO was lifted in Malaysia; it then determined the association among various demographic, personal, and clinical characteristics; COVID-19 stressors and coping; perceived social support; and depression, anxiety, and stress in these workers. Interestingly, the prevalence rates of depression (21.8%), anxiety (31.6%), and stress (29.1%) among the university healthcare workers found in our study remain within the ranges of depression (12.2–50.4%), anxiety (13.0–44.6%), and stress (29.1–71.5%) reported in healthcare workers during the peak of the COVID-19 outbreak and when the movement lockdown was in place [6,24,25,26,27]. When we compared the severity of the psychological symptoms, the prevalence rates of moderate to extremely severe depression (13.3%), moderate to extremely severe anxiety (25.8%), and moderate to extremely severe stress (8.1%) were relatively higher in our study than they were in a study of a cohort of Singaporean and Indian healthcare workers, which also used the DASS-21 as the screening tool, in response to the COVID-19 pandemic (moderate to severe depression = 5.8%, moderate to severe anxiety = 8.7%, and moderate to severe stress = 2.2%) [12]. Our findings indicate that depression, anxiety, and stress could be persistent during an infection pandemic even after the movement lockdown is lifted; this confirms the findings observed from previous infection epidemics, such as those of SARS and MERS [5].

Our findings identified two COVID-19-related stressors that significantly predisposed university healthcare workers to depression, namely, fear of frequent exposure to COVID-19 patients and agreeing that the area of living had a high prevalence of COVID-19 cases. Having more than three children was the only factor that protected against depression. Studies of healthcare workers in China during the peak of the COVID-19 outbreak reported that those who worked in medical units and those who worked as frontliners had a high risk of exposure to COVID-19 patients and fear of being infected, which predisposed healthcare workers to depression [6,24,25,28]. Moreover, those who worked in the epicenter of the COVID-19 outbreak also had a higher likelihood of developing depression [26]. Hence, our finding was in line with other studies to the effect that fear of frequent exposure to COVID-19 patients and agreeing that the area of living had a high prevalence of COVID-19 positive cases were predisposing factors for depression. In contrast, stressors related to the movement lockdown, such as frustration because of loss of daily routine during the MCO, did not predispose healthcare workers to depression. Interestingly, these findings illustrated that the fear that stems from the direct effect of the COVID-19 pandemic is independent of the psychological impact of the movement lockdown. In essence, the COVID-19 pandemic may pose a threat to the mental health of healthcare workers, independent of the effect of the movement lockdown.

Another interesting finding revealed by this study was the significant association between participants with more than three children and reduced likelihood of depression. Further questioning of those participants who had more than three children showed that they enjoyed spending time with their children, and one coping strategy that they utilized to manage their COVID-19-related work stress was to make jokes and play games with their children at home to create a fun home environment when they were not working. This finding indicated that participants who had more than three children tended to utilize affiliative and self-enhancing humor to manage their stress, which has been reported to reduce the risk of depression [29].

The only demographic characteristic predisposing participants to anxiety was being single or divorced. In addition, COVID-19-related stressors, such as fear of being frequently exposed to COVID-19 patients and not knowing the infection rate of COVID-19 in the area of living, were also predisposing factors for anxiety. In contrast, greater perceived social support from friends was protective against anxiety. Being unmarried was reported as a predisposing factor for psychological distress in a study of a cohort of 1599 people drawn from the general population in China during the peak of the COVID-19 outbreak [30]. The reasons for the association between being unmarried and anxiety indicated by this study are the lack of social commitment, feelings of loneliness under the uncertainty of the COVID-19 pandemic, and lack of sharing of the financial burden during this difficult time experienced by those who were single or divorced, which may increase their risk of anxiety [31]. Those who were fearful of frequent exposure to COVID-19-positive cases had higher odds of anxiety, which was consistent with the findings of other studies on healthcare workers [6,24,25,28]. Further questioning of the participants of what leads to the fear of frequent exposure to COVID-19 positive cases indicated that they were afraid that, if they were infected, they could be asymptomatic and unknowingly spread the infection to their family members. In addition, some participants worried that they might not have enough stock of personal protective equipment (PPE) to protect them from being infected if they were exposed to COVID-19 patients during their work assignment. These uncontrolled fears may have predisposed the participants to anxiety. Our study highlighted an interesting finding in that those who were unsure of the rate of infection in their area of living had a higher odds of developing anxiety compared with those who already knew the infection rate in their place of living. This indicated the importance of obtaining sufficient information regarding COVID-19, particularly in one’s neighborhood, as this may play a role in reducing the odds of anxiety [32,33,34].

Social support is pivotal in safeguarding mental health during an uncertain time like the COVID-19 pandemic because it may mediate reduced levels of anxiety [35,36]. Friends social support is of great importance, particularly for healthcare workers, as they work for long hours and are at risk of being infected because of their work commitment to taking care of COVID-19 patients. Hence, their main source of social support in the workplace would be from friends and colleagues. For instance, greater social support from friends allowed healthcare workers to share feelings and stressors. They could also receive more care and attention from each other [37].

Fear of frequent exposure to COVID-19 patients and having a history of pre-existing psychiatric illnesses significantly predisposed university healthcare workers to stress in our study. A higher level of perceived social support from friends was protective against stress. Muller et al. (2020) performed a rapid systematic review. Fifty-nine studies on the mental health impact of the COVID-19 pandemic on healthcare workers reported that two of the most common risk factors associated with mental health problems were exposure to COVID-19 patients and fear of being infected [38]. When we narrowed down the impact of the COVID-19 pandemic on the stress level of healthcare workers, two studies from China also indicated that exposure to infected COVID-19 patients predisposed healthcare workers to stress [24,28]. Hence, our finding that fear of exposure was significantly associated with risk of developing stress further strengthened the findings of other studies. Additional exploration of the participant views in our study illustrated that the dilemma of their obligations to manage COVID-19 patients as part of their work commitment and their fear of risking being infected and spreading the infection to their family resulting from frequent exposure to infected cases may have predisposed them to stress. People with a history of psychiatric illnesses are at risk of higher levels of stress and psychological distress. They are constantly worried about their physical health and have persistent fear of being infected during an infection pandemic or epidemic [39]. Persistent fear coupled with poor coping skills during an infection pandemic may predispose healthcare workers with pre-existing psychiatric illness to stress, as shown in the findings of this study.

Our study revealed that higher perceived social support from friends was protective not only against anxiety but also against stress. As a consequence of the work commitment of the university healthcare workers, who have been required to spend a large proportion of their time at their workplace during the COVID-19 pandemic, the main source of social support was from their colleagues and friends at their workplace. Liu et al. conducted a qualitative study on the psychological effect of 13 healthcare workers during the COVID-19 pandemic and reported that despite various difficulties experienced by these healthcare workers, such as new norms of work, fear of being infected, lack of control over patients’ situations, heavy workload and shortage of protective equipment, they were able to cope with these difficulties by utilizing the social support derived from their colleagues and friends at their workplace [40].

Based on our findings, we recommend a few points to safeguard the mental health of university healthcare workers. First, as fear of frequent exposure to COVID-19 patients predisposed these workers to depression, anxiety, and stress, university hospitals should ensure that there is a sufficient supply of PPE for use by university healthcare workers to reduce such exposure. In addition, all hospital healthcare workers should be briefed on sufficient, easy-to-follow precautionary measures and standard operating procedures to manage COVID-19 patients and prevent the spread of the infection in the workplace; this will enhance their confidence in managing COVID-19-infected patients, a task that has become a new norm and work routine. Second, as healthcare workers who were unsure of the COVID-19 situation in their area of living had increased odds of developing anxiety, university hospitals and the government should ensure the dissemination of sufficient information to university healthcare workers regarding the current COVID-19 situation in all regions of the country via university websites, blogs, and social media; this will keep healthcare workers well informed. Third, because those who lived in areas with a high prevalence of COVID-19 cases, those who were single/divorced, and those with a history of pre-existing psychiatric illnesses had increased odds of developing depression and anxiety, the higher authority of university hospitals should pay more attention to these groups of healthcare workers. Online mental health consultations and counseling or psychotherapy services should be made easily available to assist university healthcare workers who experience emotional disturbance in response to their work commitment during the COVID-19 pandemic. As psychological sequelae may persist even beyond the period of movement lockdown, these online mental health facilities should be maintained for as long as the COVID-19 pandemic persists. Fourth, as greater social support from friends may alleviate anxiety and stress, a healthcare worker support group should be initiated to allow university healthcare workers to share problems pertaining to the COVID-19 pandemic and work stress, as well as to enhance emotional support among healthcare workers. Finally, as having more children was a factor against depression, proper scheduling of duty in the workplace should be ensured to allow healthcare workers to have a sufficient amount of time for family members to maintain their mental wellbeing.

We highlight a few points to guide future research based on our findings. First, it would be interesting and important to investigate whether perceived social support has any moderating role on the relationship between COVID-19-related stressors (e.g., fear of frequent exposure to COVID-19 patients, living in areas highly prevalent for COVID-19-positive cases, and lack of sufficient information on COVID-19 situations in people’s area of living) and depression, anxiety, and stress among healthcare workers. This would further strengthen the evidence on the importance of social support in neutralizing the COVID-19 pandemic’s effect on the mental health status of healthcare workers. In fact, a systematic review of studies on the psychological impact of the COVID-19 pandemic on healthcare workers highlighted that social support is the most common protective factor against mental health complications in the COVID-19 pandemic [38]. Second, it is vital to conduct a pilot for a two-armed, double-blind, randomized control trial to evaluate the efficacy of an 8-week mindfulness-based stress reduction (MBSR) program to reduce depression, anxiety, and stress in healthcare workers by randomizing participants into an intervention group and control group. Khoury et al. (2015) conducted a meta-analysis on 29 interventional studies that investigated the efficacy of MBSR on reducing depression, anxiety, and stress in a non-clinical population and reported that MBSR has a large effect on stress and a moderate effect on depression, anxiety, psychological distress, and quality of life [41]. In addition, one strategy used by healthcare workers to cope with the COVID-19 pandemic is mindfulness [38], and an 8-week MBSR program could also be conducted online, making it suitable under the new norm of social distancing put in place to curb the spread of COVID-19. Finally, as our findings support the pivotal role of social support in lowering the likelihood of a psychological impact of the COVID-19 pandemic, it would be interesting to conduct a randomized control trial to investigate the efficacy of online social support groups for healthcare workers in terms of improving mental health status.

The findings of our study should be interpreted in view of a few limitations. First, the cross-sectional design of the present study did not allow causal inferences to be made regarding the relationship between various significant factors and depression and anxiety. Second, the use of self-reported questionnaires in this study may have led to response bias from the participants. Although we were aware of this limitation, the research team was unable to conduct in-person interviews with clinician-administered questionnaires because of the social distancing imposed by the government as a preventive measure to control the spread of COVID-19. Third, university healthcare workers were only recruited from two university hospitals in Malaysia; hence, the findings may not be generalized to reflect the entire university hospital healthcare worker population of the country. However, we selected two large university hospitals in the country for data collection, one hospital in the northern region while another in the central of Peninsular Malaysia, which may be at least fairly representative of the university healthcare worker population in Malaysia. Fourth, we did not include information on the proportion of participants who were directly involved in the care of COVID-19 patients. This may be an important confounding factor that influenced the likelihood of depression, anxiety, and stress among the participants. Finally, we did not assess the associated factors across different degrees of severity of depression, anxiety, and stress. Notwithstanding the limitations of the study, our findings provide valuable information on the mental health status of healthcare workers in Malaysia, which is still lacking.

## 5. Conclusions

This online, cross-sectional study, which investigated the mental state of university healthcare workers, revealed that the prevalence and severity of depression, anxiety, and stress remained high even after the MCO was lifted. We found that being single or divorced, fear of frequent exposure to COVID-19 patients, those who agreed that their place of living had a high prevalence of COVID-19-positive cases or being unsure of the COVID-19 situation in the area of living, and a history of pre-existing psychiatric illness predisposed participants to depression, anxiety, and stress. In contrast, greater perceived social support from friends and having more than three children were protective factors against anxiety, stress, and depression. Our study was mainly limited by the absence of information on the proportion of participants who were directly involved in the care of COVID-19 patients, and assessment of the associated factors across different degrees of severity of depression, anxiety, and stress was not performed. Despite these limitations, based on our findings, we have suggested a few practical recommendations and pinpointed a few important points for future research to help improve the mental wellbeing of healthcare workers during the COVID-19 pandemic.

## Figures and Tables

**Table 1:** Demographic and personal characteristics, clinical factors, COVID-19-related stressors, and religious coping among the participants.

Variables	*n*	%
Demographic characteristics:		
*-Age:*		
18–29 years old	47	11.8
30–45 years old	266	66.7
46–60 years old	86	21.6
*-Gender:*		
Male	107	26.8
Female	292	73.2
*-Marital status:*		
Married	308	77.2
Single/divorced	91	22.8
*-Education status:*		
Up to secondary education	45	11.3
Up to tertiary education	354	88.7
Personal characteristics:		
*-Number of children:*		
None	110	27.6
1–3 children	201	50.4
>3 children	88	22
*-Living arrangement during COVID-19:*		
Living alone	24	6
Living with friends	25	6.3
Living with family	350	87.7
Clinical factors:		
*-Pre-existing medical illnesses?*		
No	292	73.2
Yes	107	26.8
*-Pre-existing psychiatric illnesses?*		
No	388	97.2
Yes	11	2.8
COVID-19-related stressors and religious coping:		
*-Did religion help you to cope with the COVID-19 pandemic?*		
No	10	2.5
Neutral	45	11.3
Yes	344	86.2
*-Were you worried about your family during the COVID-19 pandemic?*		
No	354	88.7
Yes	45	11.3
*-Were you frustrated because of loss of daily routine during the MCO?*		
No	274	68.7
Yes	125	31.3
*-Did you feel stress because your leave was frozen during the COVID-19 pandemic?*		
No	332	83.2
Yes	67	16.8
*-Average working hours per week during the COVID-19 pandemic*	24.75 ^#^	17.55 ^$^
*-Were you afraid of being frequently exposed to COVID-19 patients?*		
No	65	16.3
Neutral	142	35.6
Yes	192	48.1
*-Were you afraid if you developed cough, flu, or fever during the COVID-19 pandemic?*	50	12.5
No	110	27.6
Neutral	239	59.9
Yes		
*-Was your area of living highly prevalent for COVID-19-positive cases?*	277	69.4
No	42	10.5
I don’t know	80	20.1
Yes		
*-Quarantine for being a close contact of COVID-19 patients:*	368	92.2
No	31	7.8
Yes		

^#^ = Mean, ^$^ = SD.

**Table 2:** Psychological characteristics and social support of the participants.

Variables	*n*	%
Depression:		
No	312	78.2
Yes	87	21.8
Severity of depression:		
None	311	77.9
Mild	34	8.5
Moderate	39	9.8
Severe	5	1.3
Extremely severe	10	2.5
Anxiety:		
No	273	68.4
Yes	126	31.6
Severity of anxiety:		
None	273	68.4
Mild	23	5.8
Moderate	72	18
Severe	14	3.5
Extremely severe	17	4.3
Stress:		
No	283	70.9
Yes	116	29.1
Severity of stress:		
None	283	70.9
Mild	84	21.1
Moderate	19	4.8
Severe	9	2.3
Extremely severe	4	1
Social support:		
Mean family support score	22.51 ^#^	4.77 ^$^
Mean friends support score	20.96 ^#^	4.56 ^$^
Mean significant others support score	22.05 ^#^	5.39 ^$^

^#^ = Mean, ^$^ = SD.

**Table 3:** Association between various factors and depression among the participants.

Variables	Crude OR ^a^	β ^b^	SE β ^b^	*p*-Value ^b^	Adjusted OR ^b^
	(95% CI)				(95% CI)
Demographic characteristics:					
*-Age:*					
18–29 years old	1				
30–45 years old	0.886 (0.434–1.811)	-	-	-	-
45–60 years old	0.519 (0.215–1.255)	-	-	-	-
*-Gender:*					
Male	1				
Female	1.196 (0.690–2.073)	-	-	-	-
*-Marital status:*					
Married	1				1
Single/divorced	2.016 (1.192–3.409) *	−0.985	0.665	0.138	0.373 (0.101–1.374)
*-Education status:*					
Up to secondary education	1				
Up to tertiary education	1.585 (0.682–3.685)	-	-	-	-
Personal characteristics:					
*-Number of children:*					
None	1				1
1–3 children	0.527 (0.311–0.891) *	−0.694	0.567	0.221	0.500 (0.165–1.517)
>3 children	0.264 (0.122–0.569) *	−1.440	0.693	0.038 **	0.237 (0.061–0.921)
*-Living arrangement during COVID-19:*					
Living alone	1				1
Living with friends	0.784 (0.241–2.549)	0.131	0.934	1.14	1.140 (0.182–7.111)
Living with family	0.417 (0.175–0.991) *	1.315	1.989	3.725	3.725 (0.076–183.681)
Clinical factors:					
*-Pre-existing medical illnesses?*					
No	1				
Yes	1.305 (0.775–2.196)	-	-	-	-
*-Pre-existing psychiatric illnesses?*					
No	1				1
Yes	6.737 (1.925–23.583) *	1.457	0.975	0.135	4.294 (0.635–29.035)
COVID-19-related stressors and religious coping:					
*-Did religion help you to cope with the COVID-19 pandemic?*					
No	1				
Neutral	2.667 (0.275–25.838)	-	-	-	-
Yes	0.968 (0.106–8.960)	-	-	-	-
*-Were you worried about your family during the COVID-19 pandemic?*					
No	1				1
Yes	2.199 (1.133–4.279) *	1.647	2.043	0.42	5.194 (0.095–284.516)
*-Were you frustrated because of loss of daily routine during the MCO?*					
No	1				1
Yes	3.756 (2.290–6.160) *	0.618	0.371	0.096	1.855 (0.897–3.838)
*-Did you feel stress because your leave was frozen during the COVID-19 pandemic?*					
No	1				
Yes	1.400 (0.767–2.556)	-	-	-	-
*-Average working hours per week during the COVID-19 pandemic*	1.006 (0.992–1.019)	-	-	-	-
*-Were you afraid of being frequently exposed to COVID-19 patients?*					
No	1				1
Neutral	2.214 (0.718–6.825)	0.284	0.601	0.636	1.329 (0.409–4.314)
Yes	7.805 (2.718–22.413) *	1.318	0.576	0.022 **	3.735 (1.208–11.551)
*-Were you afraid if you developed cough, flu, or fever during the COVID-19 pandemic?*					
No	1				
Neutral	1.096 (0.444–2.705)	-	-	-	-
Yes	1.760 (0.782–3.958)	-	-	-	-
*-Was your area of living highly prevalent for COVID-19-positive cases?*					
No	1				1
I don’t know	3.090 (1.537–6.214) *	0.731	0.486	0.133	2.078 (0.801–5.387)
Yes	2.283 (1.292–4.032) *	1.023	0.398	0.010 **	2.782 (1.275–6.068)
*-Quarantine for being a close contact of COVID-19 patients:*					
No	1				
Yes	1.272 (0.548–2.954)	-	-	-	-
Social support:					
-Mean family support score	0.554 (0.442–0.695) *	−0.072	0.056	0.197	0.930 (0.833–1.038)
-Mean friends support score	0.573 (0.453–0.724) *	−0.078	0.051	0.192	0.925 (0.836–1.023)
-Mean significant others support score	0.660 (0.543–0.801) *	−0.052	0.045	0.25	0.950 (0.870–1.037)

* = *p* < 0.1, ** = statistical significance at *p* < 0.05, ^a^ = simple logistic regression analysis: absence of depression coded as 0 (reference), presence of depression coded as 1; ^b^ = multiple logistic regression model: absence of depression coded as 0 (reference; DASS-21 Depression subscale score of 0–9), presence of depression coded as 1 (DASS-21 subscale score of ≥10); multiple logistic regression model reported Nagelkerke *R*^2^ = 0.315, *p* < 0.001, Hosmer–Lemeshow goodness-of-fit test (χ^2^ = 6.585, *df* = 8, *p* = 0.582).

**Table 4:** Associations among various factors and anxiety in the participants.

Variables	Crude OR ^a^	β ^b^	SE β ^b^	*p*-Value ^b^	Adjusted OR ^b^
	(95% CI)				(95% CI)
Demographic characteristics:					
*-Age:*					
18–29 years old	1				1
30–45 years old	0.692 (0.366–1.309)	−0.092	0.469	0.844	0.912 (0.363–2.288)
45–60 years old	0.507 (0.237–1.081) *	−0.068	0.546	0.901	0.934 (0.320–2.726)
*-Gender:*					
Male	1				
Female	1.182 (0.729–1.919)	-	-	-	-
*-Marital status:*					
Married	1				1
Single/divorced	2.151 (1.328–3.486) *	1.377	0.558	0.014 **	3.964 (1.327–11.838)
*-Education status:*					
Up to secondary education	1				
Up to tertiary education	1.154 (0.583–2.283)	-	-	-	-
Personal characteristics:					
*-Number of children:*					
None	1				1
1–3 children	0.600 (0.369–0.976) *	0.755	0.559	0.177	2.128 (0.711–6.364)
>3 children	0.481 (0.261–0.890) *	0.56	0.634	0.377	1.750 (0.505–6.064)
*-Living arrangement during the COVID-19 pandemic:*					
Living alone	1				
Living with friends	0.665 (0.211–2.090)	-	-	-	-
Living with family	0.513 (0.223–1.183)	-	-	-	-
Clinical factors:					
*-Pre-existing medical illnesses?*					
No	1				
Yes	1.138 (0.710–1.824)	-	-	-	-
*-Pre-existing psychiatric illnesses?*					
No	1				1
Yes	10.423 (2.218–48.982) *	1.4	0.969	0.149	4.053 (0.606–27.104)
COVID-19-related stressors and religious coping:					
*-Did religion help you to cope with the COVID-19 pandemic?*					
No	1				
Neutral	2.923 (0.302–28.286)	-	-	-	-
Yes	1.745 (0.193–15.797)	-	-	-	-
*-Were you worried about your family during the COVID-19 pandemic?*					
No	1				
Yes	1.687 (0.895–3.179)	-	-	-	-
*-Were you frustrated because of loss of daily routine from the MCO?*					
No	1				1
Yes	2.246 (1.440–3.505) *	0.17	0.348	0.625	1.185 (0.599–2.344)
*-Did you feel stress because your leave was frozen during the COVID-19 pandemic?*					
No	1				1
Yes	1.714 (0.999–2.941) *	0.354	0.368	0.336	1.425 (0.693–2.932)
*-Average working hours per week during the COVID-19 pandemic*	0.998 (0.986–1.010)	-	-	-	-
*-Were you afraid of being frequently exposed to COVID-19 patients?*					
No	1				1
Neutral	2.593 (0.834–5.001)	1.288	0.53	0.055	1.624 (0.683–4.233)
Yes	7.330 (3.190–17.800) *	1.465	0.535	0.006 **	4.328 (1.515–12.361)
*-Were you afraid if you developed cough, flu, or fever during the COVID-19 pandemic?*					
No	1				1
Neutral	1.340 (0.574–3.129)	0.352	0.574	0.54	1.422 (0.461–4.383)
Yes	2.851 (1.324–6.140) *	1.001	0.55	0.069	2.720 (0.925–7.998)
*-Was your area of living highly prevalent for COVID-19-positive cases?*					
No	1				1
I don’t know	3.260 (1.680–6.326) *	0.922	0.429	0.032 **	2.514 (1.085–5.827)
Yes	1.450 (0.853–2.465)	0.388	0.349	0.067	1.473 (0.743–2.921)
*-Quarantine for being a close contact of COVID-19 patients:*					
No	1				
Yes	0.878 (0.392–1.965)	-	-	-	-
Social support:					
-Mean family support score	0.612 (0.498–0.751) *	-0.033	0.049	0.502	0.967 (0.879–1.065)
-Mean friends support score	0.563 (0.451–0.701) *	−0.113	0.044	0.009 **	0.893 (0.819–0.973)
-Mean significant others support score	0.702 (0.586–0.839) *	0.003	0.041	0.946	1.003 (0.926–1.086)

* = *p* < 0.1, ** = statistical significance at *p* < 0.05, ^a^ = simple linear regression analysis: absence of anxiety coded as 0 (reference), presence of anxiety coded as 1; ^b^ = multiple logistic regression model: absence of anxiety coded as 0 (reference; DASS-21 Anxiety subscale score of 0–7), presence of anxiety coded as 1 (DASS-21 Anxiety subscale score of ≥8); multiple logistic regression model reported Nagelkerke *R*^2^ = 0.289, *p* < 0.001, Hosmer–Lemeshow goodness-of-fit test (χ^2^ = 11.470, *df* = 8, *p* = 0.176).

**Table 5:** Associations among various factors and stress in the participants.

Variables	Crude OR ^a^	β ^b^	SE β ^b^	*p*-Value ^b^	Adjusted OR ^b^
	(95% CI)				(95% CI)
Demographic characteristics:					
*-Age:*					
18–29 years old	1				
30–45 years old	1.346 (0.665–2.724)	-	-	-	-
45–60 years old	0.884 (0.387–2.016)	-	-	-	-
*-Gender:*					
Male	1				
Female	1.388 (0.836–2.304)	-	-	-	-
*-Marital status:*					
Married	1				1
Single/divorced	1.865 (1.141–3.049) *	0.383	0.573	0.505	1.466 (0.477–4.512)
*-Education status:*					
Up to secondary education	1				
Up to tertiary education	1.303 (0.636–2.670)	-	-	-	-
Personal characteristics:					
*-Number of children:*					
None	1				1
1–3 children	0.683 (0.417–1.117)	0.755	0.559	0.177	1.477 (0.518–4.211)
>3 children	0.403 (0.209–0.776) *	0.56	0.634	0.377	0.862 (0.258–2.876)
*-Living arrangement during the COVID-19 pandemic:*					
Living alone					
Living with friends	1				
Living with family	1.310 (0.418–4.107)	-	-	-	-
Clinical factors:	0.630 (0.267–1.487)	-	-	-	-
*-Pre-existing medical illnesses?*					
No	1				1
Yes	1.511 (0.941–2.425) *	0.475	0.314	0.131	1.609 (0.869–2.979)
*-Pre-existing psychiatric illnesses?*					
No	1				1
Yes	11.818 (2.513–55.583) *	2.594	1.21	0.032 **	13.382 (1.248–143.483)
COVID-19-related stressors and religious coping:					
*-Did religion help you to cope with the COVID-19 pandemic?*					
No	1				
Neutral	1.200 (0.182–7.890)	-	-	-	-
Yes	0.553 (0.091–3.361)	-	-	-	-
*-Were you worried about your family during the COVID-19 pandemic?*					
No	1				1
Yes	1.936 (1.025–3.657) *	0.199	0.53	0.707	1.220 (0.431–3.451)
*-Were you frustrated because of loss of daily routine from the MCO?*					
No	1				1
Yes	2.337 (1.487–3.675) *	−0.043	0.336	0.897	0.958 (0.496–1.850)
*-Did you feel stress because your leave was frozen during the COVID-19 pandemic?*					
No	1				
Yes	1.576 (0.909–2.734)	-	-	-	-
*-Average working hours per week during the COVID-19 pandemic*	0.996 (0.983–1.008)	-	-	-	-
*-Were you afraid of being frequently exposed to COVID-19 patients?*					
No	1				1
Neutral	1.597 (0.708–3.602)	0.432	0.466	0.354	1.541 (0.618–3.840)
Yes	4.257 (1.990–9.108) *	0.932	0.469	0.047 **	2.540 (1.014–6.364)
*-Were you afraid if you developed cough, flu, or fever during the COVID-19 pandemic?*					
No	1				1
Neutral	1.116 (0.488–2.554)	0.253	0.545	0.642	1.288 (0.442–3.753)
Yes	2.089 (0.994–4.390) *	0.771	0.511	0.132	2.162 (0.794–5.892)
*-Was your area of living highly prevalent for COVID-19-positive cases?*					
No	1				1
I don’t know	2.740 (1.411–5.323) *	0.691	0.43	0.109	1.995 (0.858–4.638)
Yes	1.536 (0.897–2.629)	0.377	0.354	0.288	1.457 (0.728–2.918)
*-Quarantine for being a close contact of COVID-19 patients:*					
No	1				1
Yes	2.148 (1.021–4.516) *	0.426	0.545	0.434	1.531 (0.526–4.452)
Social support:					
-Mean family support score	0.630 (0.513–0.773) *	−0.035	0.049	0.473	0.965 (0.876–1.063)
-Mean friends support score	0.606 (0.488–0.752) *	−0.096	0.044	0.029 **	0.909 (0.834–0.990)
-Mean significant others support score	0.727 (0.607–0.871) *	−0.018	0.04	0.212	0.982 (0.908–1.062)

* = *p* < 0.1, ** = statistical significance at *p* < 0.05, ^a^ = absence of stress coded as 0 (reference), presence of stress coded as 1; ^b^ = absence of stress coded as 0 (reference; DASS-21 Stress subscale score = 0–14), presence of stress coded as 1 (DASS-21 Stress subscale score ≥ 15), multiple logistic regression model reported Nagelkerke *R*^2^ = 0.249, *p* < 0.001, Hosmer–Lemeshow goodness-of-fit test (χ^2^ = 4.781, *df* = 8, *p* = 0.781).
